# Macrophage polarization in rheumatoid arthritis: signaling pathways, metabolic reprogramming, and crosstalk with synovial fibroblasts

**DOI:** 10.3389/fimmu.2024.1394108

**Published:** 2024-05-10

**Authors:** Yixin Zheng, Kai Wei, Ping Jiang, Jianan Zhao, Yu Shan, Yiming Shi, Fuyu Zhao, Cen Chang, Yunshen Li, Mi Zhou, Xinliang Lv, Shicheng Guo, Dongyi He

**Affiliations:** ^1^ Department of Rheumatology, Shanghai Guanghua Hospital of Integrative Medicine, Shanghai University of Traditional Chinese Medicine, Shanghai, China; ^2^ Guanghua Clinical Medical College, Shanghai University of Traditional Chinese Medicine, Shanghai, China; ^3^ Institute of Arthritis Research in Integrative Medicine, Shanghai Academy of Traditional Chinese Medicine, Shanghai, China; ^4^ Department of Rheumatology, Traditional Chinese Medicine Hospital of Inner Mongolia Autonomous Region, Hohhot, Inner Mongolia Autonomous Region, China

**Keywords:** rheumatoid arthritis, inflammation, macrophages, macrophage polarization, fibroblasts

## Abstract

Rheumatoid arthritis (RA) is a chronic autoimmune disease characterized by persistent synovial inflammation and progressive joint destruction. Macrophages are key effector cells that play a central role in RA pathogenesis through their ability to polarize into distinct functional phenotypes. An imbalance favoring pro-inflammatory M1 macrophages over anti-inflammatory M2 macrophages disrupts immune homeostasis and exacerbates joint inflammation. Multiple signaling pathways, including Notch, JAK/STAT, NF-κb, and MAPK, regulate macrophage polarization towards the M1 phenotype in RA. Metabolic reprogramming also contributes to this process, with M1 macrophages prioritizing glycolysis while M2 macrophages utilize oxidative phosphorylation. Redressing this imbalance by modulating macrophage polarization and metabolic state represents a promising therapeutic strategy. Furthermore, complex bidirectional interactions exist between synovial macrophages and fibroblast-like synoviocytes (FLS), forming a self-perpetuating inflammatory loop. Macrophage-derived factors promote aggressive phenotypes in FLS, while FLS-secreted mediators contribute to aberrant macrophage activation. Elucidating the signaling networks governing macrophage polarization, metabolic adaptations, and crosstalk with FLS is crucial to developing targeted therapies that can restore immune homeostasis and mitigate joint pathology in RA.

## 1 Introduction

Rheumatoid arthritis (RA) is a chronic autoimmune disorder marked by inflammation of synovial tissue, destruction of cartilage and bone, affecting approximately 1% of the population and imposing significant burdens on individuals and society (1–3). Timely diagnosis and intervention are essential in preventing disease progression and enhancing treatment outcomes for RA patients (4). However, the pathological mechanism of RA is not fully understood. Synovial macrophages are associated with the severity of RA and cartilage damage (5). Macrophages, as versatile cells, play a crucial role in tissue remodeling and repair by eliminating invasive pathogens and senescent cells, thereby safeguarding the human body from infections, injuries, and cancer (6). These cells originate from various lineages during development and maintain their diversity into adulthood (7, 8). Following pathogen infiltration or tissue injury, tissue-resident macrophages typically transition to an activated or inflammatory state, a phenomenon referred to as macrophage polarization (9). These cells are commonly called macrophages (M1) and alternatively activated macrophages (M2) types (10, 11). M1 macrophages that mediate resistance to pathogens and tissue destruction by produce pro-inflammatory cytokines like tumor necrosis factor (TNF), Interleukin (IL)-6 (IL-6) and IL-1β, C-C motif chemokine ligand 2 (CCL2), IL-8, IL-12 and IL-23 (12, 13). M2 macrophages can remove debris and promote tissue repair by produce anti-inflammatory cytokines consisting of transforming growth factor-β (TGF-β), IL-10, IL-4, IL-13 (14, 15). Exploring the polarization of macrophages in RA is a hot field (16). The abnormal immune microenvironment in RA patients promotes metabolic reprogramming, alters macrophage polarization, disrupts the dynamic balance of M1 and M2 macrophages, and promotes tissue inflammation. Macrophages exist in every tissue of the human body and exhibit anatomical and functional diversity (9). There is an imbalance of M1/M2 in the synovial fluid (17), synovium (18), and peripheral blood (15) tissues of RA patients. Macrophages produce a large amount of pro-inflammatory cytokine TNF. Current treatment options such as disease-modifying antirheumatic drugs and monoclonal antibodies targeting TNF blockade can alleviate some of the effects on macrophage activation, but have no specificity for macrophages. At present, there is no therapy that has been proven to be effective and safe in specifically eliminating RA macrophages. Deciphering how the process of macrophage polarization and functions affect RA may provide landscape for the targeted therapy.

## 2 Macrophage polarization in RA

Research indicates a positive correlation between macrophage abundance and the extent of synovial hyperplasia, as well as a direct relationship with disease activity as measured by DAS28 scores and joint erosion in patients (19). Notably, M1 macrophages are predominantly present in individuals with active rheumatoid arthritis (RA), whereas M2 macrophages are associated with lower disease activity or clinical remission in RA patients (20). Macrophage polarization is intricately regulated by a variety of signaling pathways, such as notch signaling pathway (Notch), Janus kinase/signal transducer and activators of transcription (JAK/STAT), mitogen-activated protein kinase (MAPK), and nuclear factor kappa-B (NF-κb) pathways. Specifically, AKT, p65/p50, p38, NF-κB, and AP-1 are associated with M1 polarization, while SMAD3, p50/p50, and SMADs are associated with M2 polarization (21, 22).

Macrophages are acknowledged as key contributors to the production of inflammatory mediators, including TNF-α, IL-6, and IL-1β, which are essential in initiating the inflammatory cascade. Furthermore, these mediators can also promote macrophage polarization towards the M1 phenotype through diverse signaling pathways. In RA, the transcription factor STAT3 plays a crucial role in directing macrophage polarization towards the M1 phenotype (23), with IL-6 serving as the primary activator of STAT3 in this context (24). The activation of STAT3 by IL-6 contributes to joint destruction in RA by promoting the upregulation of receptor activator of nuclear factor kappa-B ligand (RANKL) in osteoblasts and facilitating the differentiation of osteoclasts (25). The activation of the NF-κB signaling pathway by TNF-α and IL-1 results in the phosphorylation and dissociation of the IκBα/NF-κB complex (26). In RA, NF-κB activation facilitates M1 polarization, ultimately resulting in the secretion of a substantial quantity of mature inflammatory cytokines (27). Additionally, activation of Toll-like receptor 4 (TLR4) induces NF-kB signaling in M1macrophages, resulting in the secretion of IL-6, TNF-α, and IL-1β in synovial macrophages of patients with RA (28). In RA, the pro-inflammatory cytokines TNF-α, IL-1 β, and IL-6 stimulate the activation of the MAPK signaling pathway by inducing phosphorylation of ERK1/2, JNK, and p38 kinases in synovial cells (29). This activation of the stress-activated protein kinases (SAPK)/MAPK pathway by pro-inflammatory cytokines in RA leads to enhanced macrophage proliferation and survival (30). Furthermore, the proteins c-Fos and c-Jun exhibit elevated levels of expression in RA synovial tissue and play a role in the polarization of macrophages (31, 32). Specifically, c-Fos functions by directly suppressing the expression of Arginase (Arg) 1 in macrophages, thereby diminishing their anti-inflammatory properties. On the other hand, c-Jun promotes the upregulation of cyclooxygenase-2 (Cox-2) in macrophages, suppresses Arg1 expression, and influences the polarization of macrophages towards the M1 subtype.

The polarization and transformation of macrophages can contribute to the exacerbation of joint inflammation in RA (33). Throughout the progression of RA, numerous factors can disrupt the delicate balance between M1 and M2 macrophages, leading to an increase in M1 macrophages and subsequently intensifying the inflammatory response in RA (17, 34, 35). Recent research has indicated that natural medicines, such as traditional Chinese medicine, along with nano formulations, have the ability to modulate M1 to M2 repolarization through pathway signaling and antioxidant properties, thereby mitigating joint inflammation and tissue damage in RA.

### 2.1 M1 macrophage polarization worsens RA by releasing inflammatory factors

The signaling pathways implicated in the polarization of macrophages towards the M1 phenotype are extensively documented in the literature. Previous research has identified key pathways associated with inflammation mediated by M1 macrophages, such as the Notch, ERK, MAPK, JAK/STAT, and MAPK signaling pathways.

Notch signaling appears to be a causative factor in the imbalance between M1 and M2 macrophages in RA, thus playing a significant role in the pathogenesis of RA. Sun et al. (36) observed that M1 macrophages derived from bone marrow (BM) exhibit activated Notch signaling in the inflamed joints of TNF-α-transgenic mice. Furthermore, they found that RA synovial tissue promotes the activation of Notch signaling in BM-derived macrophages, leading to M1 polarization. Treatment with thapsigargin, a Notch inhibitor, reduces TNF-α-induced M1 macrophage polarization and mitigates inflammation and joint bone loss by promoting a switch from M1 to M2 macrophages. The Notch signaling pathway is crucial in regulating osteoclast differentiation and bone-resorbing activity by directly influencing osteoclast precursors, as well as indirectly affecting cells of the osteoblast lineage and immune system (37). In joints affected by RA, Notch1 is upregulated and activated in fibroblast-like synoviocytes, Th17 cells, and M1 macrophages, promoting the secretion of pro-inflammatory cytokines such as TNF-α, IL-6, and IL-17. This cascade of events ultimately results in inflammation, bone degradation, and joint bone loss (38). The ERK signaling pathway, a member of the MAPK family, plays a significant role in regulating macrophage phenotype (39). Activation of the ERK1/2 pathway during LPS-induced inflammatory responses can lead to M1 polarization and suppression of inflammatory reactions (40). Nesfatin-1 induces c-c motif chemokine ligand 2 (CCL2) overexpression through the MEK/ERK pathway in RA synovial fibroblast, in which overexpressed CCL2 enhanced the polarization of M1 macrophages by treating THP-1-derived (M0) macrophages with synovial fibroblast conditioned medium (41). Nesfatin-1 identified as a potential risk factor for RA (42, 43). CCL2 levels are high in RA synovial tissue (44), which induces the recruitment and migration of monocytes to inflammatory sites in arthritis (45), thereby facilitating the progression of knee synovitis in affected individuals (46). The JAK-STAT pathway is stimulated by diverse inflammatory stimuli, influencing the differentiation of macrophages and the inflammatory response. Specifically, the activation of the JAK/STAT1 signaling cascade by IFN- γ facilitates the release of pro-inflammatory mediators by M1 macrophages. STAT1 activity is conducive to M1 polarization and its inhibition can lead to M2 polarization (47, 48).

### 2.2 Repolarizing macrophages from M1 to M2 can help relieve RA

Natural products, such as extracts derived from traditional Chinese medicine, have the potential to effectively manage RA by modulating the repolarization of M1 to M2 macrophages. Specifically, Tripterygium wilfordii glycosides have been shown to suppress the secretion of pro-inflammatory cytokines (IL-1, IL-6, CXCL8, TNF-α, and VEGF-A) by M1 macrophages while concurrently enhancing the expression of the anti-inflammatory cytokine IL-10 in M2 macrophages. The results of liquid phase chip quantitative analysis demonstrate that Triptolide decreases TNF in arthritis models, as well as levels of α, CXCL2, and VEGF, while increasing levels of IL-4 and IL-10. Additionally, Triptolide A inhibits NF- κB, PI3K/AKT, and p38 MAPK signaling pathways, thereby ameliorating RA joint inflammation (49). Wuweiganlu (WGL) is a renowned formulation primarily utilized for the management of RA and other chronic conditions as recommended by Tibetan medicine. *In vitro* experiments have shown that WGLWE induces the polarization of M1 macrophages towards the M2 phenotype, while also inhibiting the secretion of proinflammatory cytokines TNF-α and IL-6 (50).

In addition to pro-inflammatory cytokines, the pathogenesis of RA encompasses a multitude of pathological factors that synergistically contribute to the perpetuation of the inflammatory response and exacerbation of tissue damage. Metabolically, M1 macrophages predominantly rely on aerobic glycolysis, whereas M2 macrophages predominantly utilize oxidative phosphorylation (51). In the setting of joint inflammation, the formation of synovial pannus and the establishment of hypoxic inflammatory microenvironments significantly enhance the glycolytic metabolism of macrophages, driving their polarization towards the M1 phenotype. HIF-1α, NF-κB, Notch-1, and JAK-STAT have been identified as factors influencing alterations in macrophage metabolic phenotype. Of particular note, the activation of HIF-1α in macrophages has emerged as a crucial signaling mechanism governing aerobic glycolysis and M1 polarization in recent research (52). Inhibition of HIF-1 in macrophages suppresses glycolysis levels and M1 polarization, as well as impairs cell migration and bactericidal function (53, 54). The PI3K-AKT-mTOR-HIF-1α pathway serves as the fundamental mechanism for improving angiogenesis in RA (55). In comparison to synovial macrophages in healthy control groups, macrophages in RA synovium exhibit heightened expression of HIF-1 α (56), leading to enhanced transcription of glycolytic enzymes and upregulation of critical pro-inflammatory cytokines like IL-1 β (57). The accumulation of succinate, an intermediate metabolite of the tricarboxylic acid (TCA) cycle, is hypothesized to induce the activation of HIF-1 α, subsequently resulting in the generation of downstream IL-1 β (57). Recent studies have demonstrated that the reduction of citrate in M1 polarized macrophages (58) and the blockade of HIF-1α-related signaling pathways (59) can effectively suppress the overactivation of glycolytic metabolism in M1 cells. Additionally, the activation of the AMPK pathway results in the reprogramming of M1 glycolysis (60), inhibition of mTORC1 activity, suppression of protein synthesis, regulation of macrophage glucose metabolism and proliferation, and enhancement of mitochondrial enzyme activity to facilitate oxidative phosphorylation (61). Besides, lysine acetyltransferase 2A in synovial tissue promotes macrophage glycolytic reprogramming by inhibiting the activity of nuclear factor-erythroid 2-related factor 2 and downstream antioxidant molecules (62). These biological processes ultimately result in a phenotypic transition from M1 to M2.

## 3 Synovial macrophage and fibroblast crosstalk during RA pathogenesis

The synovial membrane typically consists of two distinct layers: the inner lining layer and the sub-lining layer (63). The inner lining layer, composed of synovial lining cells (SLCs), is further categorized into type A (macrophage-like) and type B (fibroblast-like) cells (64), which originate from bone marrow mononuclear phagocytes and mesenchymal stem cells, respectively (65). Among the cells comprising the synovial lining layer, macrophages represent approximately macrophage-like synoviocytes (MLS) represent approximately 20% while fibroblasts-like synoviocytes (FLS) make up the remaining 80% (66, 67). Macrophage-like cells demonstrate a highly activated phenotype and secrete numerous pro-inflammatory cytokines, chemokines, and growth factors, which in turn stimulate local fibroblast-like synovial cells (FLS) to produce IL-6, prostaglandins, and matrix metalloproteinases (MMPs). This cascade establishes a paracrine/autocrine network that perpetuates synovitis and leads to continuous degradation of the extracellular matrix (68, 69).

### 3.1 MLS regulates FLS phenotypes in RA

In recent years, research has demonstrated that macrophages can modulate the phenotype of FLS via diverse biological mechanisms, including post-translational protein modifications. This highlights the regulatory role of macrophages in controlling the phenotype of FLS. The post-translational modifications of malondialdehyde acetaldehyde (MAA) and citrulline (CIT) are implicated in the pathogenesis of RA (RA). Upon modification by MAA and/or citrullinated fibrinogen, macrophages secrete soluble platelet-derived growth factor (PDGF) PDGF-BB subtypes, which can promote fibroblast-like synoviocyte (FLS) differentiation into invasive phenotypes. Compared to the control group, there is an upregulation of the expression of phosphorylated c-Jun N-terminal kinase (p-JNK), phosphorylated extracellular signal-regulated kinase 1/2 (p-Erk1/2), phosphorylated protein kinase B (p-Akt) signaling pathways, as well as vimentin (VIM) and type II collagen (COL2A1), indicating an increase in mRNA expression of these genes (70). Extracellular traps (ET) are composed of histones, double-stranded DNA, myeloperoxidase, or elastase and are considered the primary source of citrullinated autoantigens in RA, leading to the production of anti-citrullinated protein antibodies (ACPA). Macrophages produce a specific type of ET known as macrophage extracellular trap (MET) (71). The stimulation of the DNA sensor GMP-AMP synthase (cGAS) in RA RA-FLS by macrophage-derived microvesicles (METs) from HP-1 cells triggers the activation of the PI3K/Akt signaling pathway, leading to enhanced proliferation, migration, invasion, and expression of inflammatory cytokines in RA-FLS. The findings indicate a significant upregulation of proinflammatory cytokines such as TNF and IL-1β, as well as matrix-degrading enzymes MMP-9 and MMP-13, in MET-stimulated RA-FLS compared to untreated controls (72). As single-cell sequencing and transcriptome techniques have advanced in the study of RA (RA), numerous investigations have explored the heterogeneity of macrophage subtypes, indicating that macrophage polarization extends beyond the traditional M1 and M2 classifications. Through extensive single-cell RNA sequencing (scRNA seq) analysis, thorough phenotypic, spatial, and functional assessments, Alivernini S et al. (73) identified two distinct populations within the macrophage-like synoviocytes (MLS), further stratified into nine clusters with distinct characteristics. The MerTK^neg^CD206^neg^ cluster elicits the production of pro-inflammatory cytokines and instigates inflammatory responses in FLS. The MerTK^pos^CD206^pos^ cluster in RA patients is associated with the production of lipid mediators during the continuous remission phase of the disease, which resolves inflammation and promotes the repair phenotype of FLS. This interaction between the MerTK^pos^CD206^pos^ cluster and FLS in the remission phase of RA plays a crucial role in maintaining joint immune homeostasis.

### 3.2 FLS regulates MLS phenotypes in RA

FLS has the capacity to induce macrophage polarization through distinct mechanisms, diverging from the traditional M1/M2 polarization paradigm. Prolonged exposure to pro-inflammatory conditions may result in heightened prostaglandin (PGE2) production in FLS. In conjunction with inflammatory mediators, macrophages have a tendency to polarize towards heparin-bound EGF-like growth factors (HBEGF) that deviate from the traditional M1 and M2 polarization phenotypes. The presence of HBEGF-enriched inflammatory macrophages in rheumatoid arthritis has been shown to give rise to distinct subpopulations of inflammatory mediators, including IL-1 and the growth factor HB-EGF, as well as surface embryonic protein. These macrophages also exhibit heightened expression of pro-inflammatory genes such as IL1B and CXCL2. Pathway analysis indicates that FLS may influence the metabolic profile of macrophages treated with TNF, resulting in a collective suppression of factors involved in oxidative phosphorylation. On the other hand, it has been observed that HBEGF inflammatory macrophages have the ability to stimulate the invasiveness of FLS, whereas EGFR inhibitors, originally designed for cancer treatment, have shown efficacy in inhibiting the fibroblast responses induced by macrophages in RA tissue (74). Extracellular vesicles (EVs) are small membrane-bound particles released by cells into the extracellular space, containing a variety of biomolecules such as RNA, lipids, proteins, and DNA. These EVs have the ability to transfer their contents to recipient cells, influencing their phenotype. Exosomes, a subtype of EVs with a diameter of ≤ 100-150 nm, are formed within multivesicular bodies. The PTX3 protein found in exosomes derived from RA-FLS does not impact the mRNA expression of TNF, IL6, and IL1B in M1 macrophages. However, experimental results from transwell migration assays indicate that PTX3 can enhance macrophage migration (75). PTX3 is an innate immune inflammatory modulator consisting of lipopolysaccharide, IL-1β, TNF-α, and other inflammatory factors. These components are associated with processes such as angiogenesis, atherosclerosis, cell proliferation, and tumor evasion (76). Furthermore, research conducted on animals has demonstrated that the interaction between MLS and FLS in inflammatory environments can trigger metabolic alterations, enhancing the longevity of MLS and contributing to the development of chronic inflammation in RA (77). Separate mouse arthritis tissue-derived synovial macrophages (ADSM) and arthritis tissue-derived synovial fibroblasts (ADSF), and culture ADSM in serum-free conditioned medium of ADSF. The expression levels of inflammatory macrophage markers Nos2, Tnf, Il-1b, and CD86 were significantly increased in ADSM. Metabolic flux analysis revealed upregulation of glycolysis and mitochondrial respiration in ADSMd, suggesting a potential long-lived phenotype in ADSM.

The crosstalk between MLS and FLS has been shown to impact the pathogenesis of RA by promoting bone destruction (78). Additionally, the interaction between macrophages and fibroblasts plays a role in mediating cartilage degradation and facilitating the migration of pathogenic osteoclast precursors to the inflamed synovium. Endothelial cells and synovial fibroblasts are significant sources of CX3CL1, a chemokine that has been implicated in these processes. Recent research has identified a specific subset of macrophages, characterized by high expression of CX3CR1 and low expression of Ly6C, F4/80, and I-A/I-E, known as arthritis-associated osteoclast macrophages (AtoMs), as a precursor population of pathogenic osteoclasts in arthritis. The bidirectional signal is produced between synovial macrophages and fibroblasts via the interaction of CX3CR1 CX3CL1. However, the precise mechanism remains unclear (79). Li and colleagues (80) conducted an examination of the influence of CCR2 expression on RA-FLS by co-culturing them with macrophages in an *in vitro* model. The results showed that treatment of RA-FLS with a CCR2 antagonist led to reduced expression of IL-1, IL-6, and TNF-α in macrophages, as well as induction of M2-type differentiation. The specific mechanism underlying these effects, potentially involving the downregulation of inflammatory cytokines and matrix metalloproteinases in RA-FLS, remains unclear.

## 4 Conclusion

**Figure 1:**
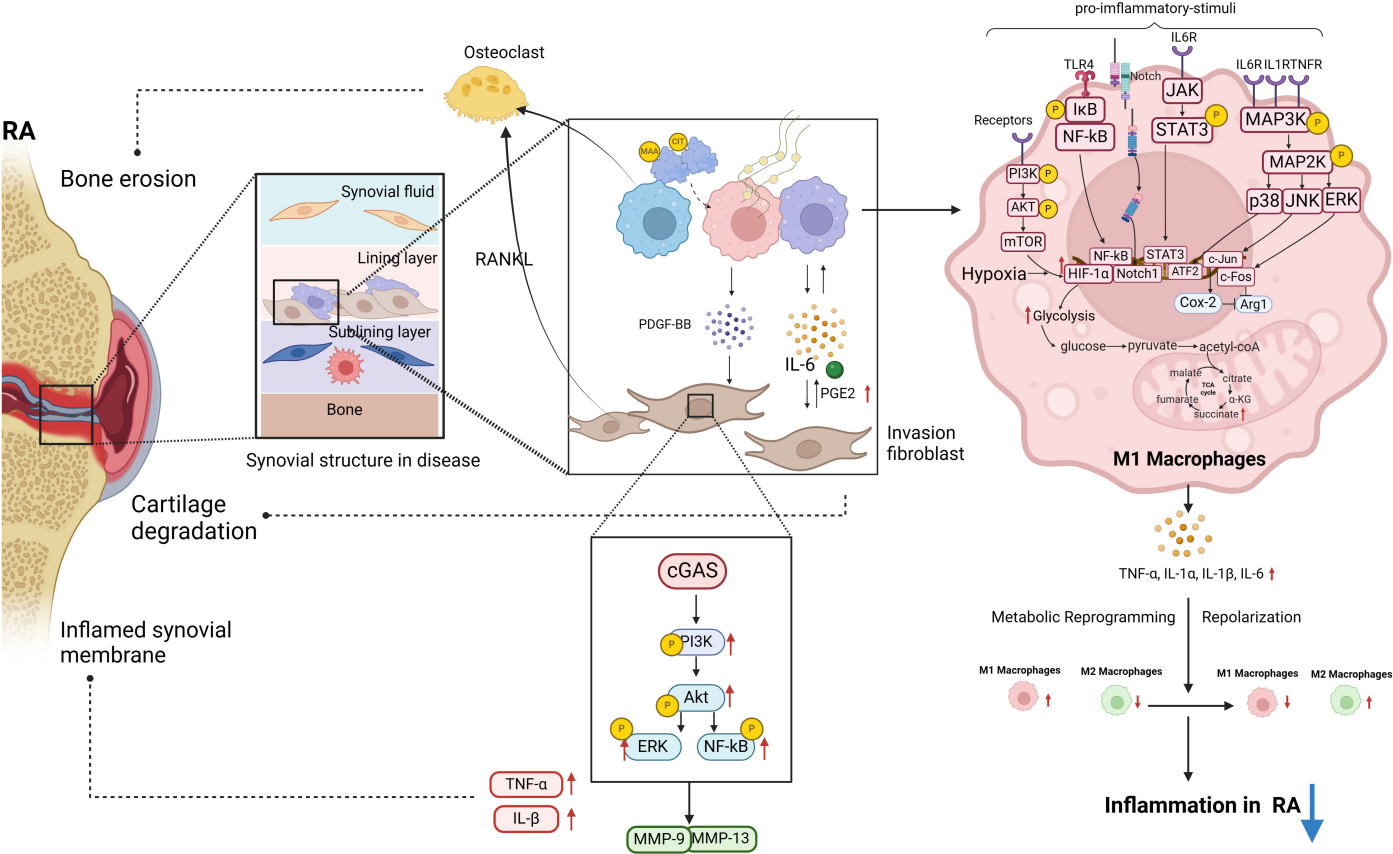
The polarization of macrophages and their intercellular communication with fibroblasts play a significant role in the pathogenesis of RA. Post-translational modifications of MAA and CIT are linked to RA pathogenesis. MAA and citrullinated fibrinogen can lead to macrophages secreting PDGF-BB, promoting invasive FLS differentiation. In RA-FLS cells, stimulation of cGAS by METs from HP-1 cells activates the PI3K/Akt signaling pathway, resulting in enhanced proliferation, migration, invasion, and expression of inflammatory cytokines. A significant upregulation of proinflammatory cytokines and matrix-degrading enzymes MMP-9 and MMP-13 was observed in stimulated RA-FLS. This diagram illustrates the intracellular signaling pathways triggered by RA pro-inflammatory M1 macrophages in the pathogenesis of RA, along with the M1 glycolytic metabolism process. These pathways within M1 macrophages are essential in driving RA inflammation (biorender.com).

In this review, we aimed to elucidate the signaling networks that regulate macrophage polarization, metabolic adaptations, and interactions with FLS in order to develop targeted therapies that can restore immune homeostasis and alleviate joint pathology in rheumatoid arthritis (**Figure 1**). In RA, macrophages that are excessively activated upregulate the expression of toll-like receptors, leading to the initiation of synovitis and cartilage degradation through the secretion of chemokines, pro-inflammatory cytokines, and proteolytic enzymes. It has been suggested that the selective elimination of synovial inflammatory macrophages in RA can be achieved through the use of CD64-directed immunotoxins (81). In RA patients, inflammatory macrophages in synovial fluid exhibit elevated levels of CD64 compared to monocytes in peripheral blood, making them potential targets for selective elimination via apoptotic cell death (81). Despite current efforts to target macrophages by modulating their phenotypes, there are currently no specific drugs available for this purpose (82). In individuals with RA, the aberrant immune microenvironment facilitates metabolic reprogramming, modulates macrophage polarization, disturbs the equilibrium between M1 and M2 macrophages, and hinders tissue inflammation through intricate mechanisms. Investigating the intercellular communication and interaction between diverse cell types, such as synovial fibroblasts and synovial macrophages, may aid in restoring the dysregulation of macrophage M1/M2 balance. Inhibiting M1 macrophage polarization and inducing M2 macrophage polarization have been identified as promising approaches for drug development in the treatment of RA. Current treatment modalities for RA are constrained by issues such as frequent dosing, limited bioavailability, transient efficacy, and significant long-term side effects. Consequently, researchers have recently focused on the utilization of bioactive nanoparticles and macrophage-derived large vesicle-coated nanoparticles to address these challenges.

The overstimulation of macrophages resulting in the production of inflammatory mediators has been a prominent focus of research in the pathogenesis of rheumatoid arthritis. While significant progress has been made in understanding the roles of macrophage polarization and macrophage-fibroblast crosstalk in RA pathogenesis, several key challenges remain. Defining the full spectrum of macrophage phenotypes beyond the simplistic M1/M2 categorization is an area of active research, as single-cell studies reveal a high degree of heterogeneity. Unraveling the complex interplay between signaling pathways, epigenetic modifications, and metabolic adaptations that shape macrophage identity represents another major hurdle. Developing therapeutic strategies to precisely modulate these processes *in vivo* without off-target effects poses an additional obstacle. Furthermore, the bidirectional nature of macrophage-fibroblast communication underscores the need for a holistic examination of the RA synovial microenvironment. Future efforts should focus on mapping the spatiotemporal dynamics of these cellular interactions at single-cell resolution, deciphering mechanisms of intercellular signal integration, and evaluating the therapeutic impact of simultaneously targeting both macrophages and fibroblasts. Multidisciplinary approaches combining advanced techniques like spatial transcriptomics, computational modeling, and targeted nanoparticle engineering may pave the way towards precision immunomodulatory interventions tailored to individual RA patients.

## Author contributions

YZ: Data curation, Writing – original draft, Writing – review & editing. KW: Data curation, Writing – review & editing. PJ: Data curation, Writing – review & editing. JZ: Data curation, Writing – review & editing. YuS: Data curation, Writing – review & editing. YiS: Data curation, Writing – review & editing. FZ: Data curation, Writing – review & editing. CC: Data curation, Writing – review & editing. YL: Data curation, Writing – review & editing. MZ: Data curation, Writing – review & editing. XL: Data curation, Writing – review & editing. SG: Conceptualization, Writing – review & editing. DH: Writing – review & editing, Conceptualization.
